# Oil palm plantations in Peninsular Malaysia: Determinants and constraints on expansion

**DOI:** 10.1371/journal.pone.0210628

**Published:** 2019-02-20

**Authors:** Varada S. Shevade, Tatiana V. Loboda

**Affiliations:** Department of Geographical Sciences, University of Maryland, College Park, Maryland, United States of America; James Cook University, AUSTRALIA

## Abstract

Agricultural expansion is one of the leading causes of deforestation in the tropics and in Southeast Asia it is predominantly driven by large-scale production for international trade. Peninsular Malaysia has a long history of plantation agriculture and has been a predominantly resource-based economy where expanding plantations like those of oil palm continue to replace natural forests. Habitat loss from deforestation and expanding plantations threatens Malaysian biodiversity. Expanding industrial plantations have also been responsible for drainage and conversions of peatland forests resulting in release of large amounts of carbon dioxide. The demand for palm oil is expected to increase further and result in greater pressures on tropical forests. Given Malaysia’s high biophysical suitability for oil palm cultivation, it is important to understand patterns of oil palm expansion to better predict forest areas that are vulnerable to future expansion. We study natural forest conversion to industrial oil palm in Peninsular Malaysia between 1988 and 2012 to identify determinants of recent oil palm expansion using logistic regression and hierarchical partitioning. Using maps of recent conversions and remaining forests, we characterize agro-environmental suitability and accessibility for the past and future conversions. We find that accessibility to previously existing plantations is the strongest determinant of oil palm expansion and is significant throughout the study period. Almost all (> 99%) of the forest loss between 1988 and 2012 that has been converted to industrial oil palm plantations is within 1 km from oil palm plantations that have been established earlier. Although most forest conversions to industrial oil palm have been in areas of high biophysical suitability, there has been an increase in converted area in regions with low oil palm suitability since 2006. We find that reduced suitability does not necessarily restrict conversions to industrial oil palm in the region; however, lack of access to established plantations does.

## Introduction

Agricultural expansion is one of the primary causes of deforestation globally [1–3]. Between 1980 and 2000 about 83% of agricultural expansion in the tropics occurred on previously forested land [2]. With increasing urban populations and greater consumption of agricultural products [4,5], over time, the primary agents of deforestation resulting from agricultural expansion have changed from state-enabled smallholders in the 1980s to large enterprises producing for international markets in the 1990s, particularly in the Amazon and Southeast Asia [4]. The amplified global trade of commodities has linked distant areas of consumption and production and has lead to local environmental impacts at sites producing goods for meeting international demand [6]; for instance road building and forest clearing has also become enterprise-driven [4] and trade of agricultural commodities has been linked specifically to high rates of forest loss in the humid tropics [5]. Commercial actors producing for international markets are now globally the most important drivers of deforestation in developing countries and contribute to approximately 35% of the deforestation in Asia [3].

Between 2000 and 2012 Malaysia had the highest percent of tree cover loss relative to its land area [7]. Deforestation and expanding plantations, especially those of oil palm, are a threat to biodiversity in Malaysia, which had the highest number of threatened species per square kilometer in relation to palm oil production in 2005 [8]. Habitat loss from expanding plantations particularly threatens megafauna like the tiger, the rhino and the elephant in Peninsular Malaysia [9], where expanding plantations continue to replace natural forests and an estimated 651,757 ha of forest loss between 1988 and 2012 had been converted to plantations by 2014 [10]. Owing to their uniform tree-age structure and homogeneity compared to forests, oil palm plantations support fewer species [11, 12]. Conversion of peatswamp forests to oil palm in Peninsular Malaysia by early 2000s was estimated to have caused a loss of 46 species of forest birds [13].

In Peninsular Malaysia, agricultural expansion and extension of permanently cultivated land has always been the major cause of deforestation or forest conversion [14]. Malaysia has been a predominantly resource-based economy with its exports dependent on minerals, timber, and other tree crops such as rubber and oil palm [14]. While major drivers of deforestation switched from logging for export (1950–1980) to plantation expansion during the 1980-1990s [15], large industrial plantations are well established in the region. First large industrial plantations appeared in the region during the early 1900s and have since expanded with the increased demand for commodities. In the early phase of industrial plantations, rubber plantations spread with increasing rubber prices early in the 20^th^ century. Following a drop in rubber prices during the 1960s, an increased demand for palm oil eventually led to the growth of oil palm plantations; including both development of new plantations on previously forested land and those replacing old rubber plantations. Oil palm plantations accounted for the largest area under industrial tree crops in Malaysia by 1990 [16] and by 2010 oil palm plantations covered 20% of Peninsular Malaysia [17]. A significant portion of the expanding plantations have been established by converting natural forests, however, estimates for oil palm expansion originating from deforestation vary drastically. Countrywide estimates for the proportion of expanding oil palm replacing forests in Malaysia range from 38–39% between late 1980s or 1990 and 2010 [17, 18] to 55–59% during 1990–2005 [19].

The patterns and extent of plantation expansion driven deforestation vary regionally and temporally. The countrywide proportion of new oil palm plantations established by converting forests reduced from ~56% during 1990–2000 to ~33% during 2006–2010 [17]. Direct conversion of forests to plantations has been more common in Sabah and Sarawak (Malaysian Borneo) [17, 20]. Forest conversions during 1990–2000 accounted for 62% and 48% of new oil palm plantations in Sabah and Sarawak respectively, while in Peninsular Malaysia, an estimated 28% of oil palm expansion during 1990–2010 originated from forest conversion [17] and the proportional contribution of forest conversions has reduced from ~38% in 1990–2000 to ~ 6% during 2006–2010 [17].

The global importance of palm oil has risen to new levels since the beginning of the 21^st^ century; by 2007, palm oil constituted 30% of global production of vegetable oils while palm oil exports accounted for 60% of global exports in oils and fats by volume [21]. Malaysia is currently the second largest producer [22] and one of the largest exporters of palm oil products accounting for 44% of global palm oil exports (Malaysian Palm Oil Council) [23]. The Malaysian palm oil industry plays a significant role in the Malaysian economy and in 2015 oil palm contributed to 4.17% of Malaysia’s Gross Domestic Product [24]. Oil palm plantations covered 5.2 Mha in Malaysia in 2010 with 2.7 Mha in Peninsular Malaysia and the remaining in Malaysian Borneo [17]. Additionally, between 48–56% of all deforestation in Peninsular Malaysia has been converted to oil palm plantations in the 1990s and 2000s [10, 17]. Peninsular Malaysia has also continued to produce a large fraction of Malaysia’s total palm oil products; around 52% of Malaysia’s crude palm oil was produced by Peninsular Malaysia in 2017 [25]. As the demand for palm oil is expected to grow [26], up to 5 Mha of additional land would be required to meet Malaysia’s production projections for 2020 [22]. Given this anticipated demand [26] and the recent trends of forest conversion [10,17], Malaysia’s remaining forests continue to be threatened by expanding oil palm plantations. Understanding factors that influence expansion of plantations is thus important to identify where future plantations might be established.

Commodity crop expansion pathways are controlled by availability of suitable forestland versus other land areas, relative economic and technical characteristics of different land uses, differences in constraints and opportunities for small and large-scale actors, and factors affecting costs versus benefits of forest clearing [27]. Typically, land cover and land use changes are modeled following land rent theories that use factors determining costs and returns of land conversion to vary with potential profitability from that conversion [28–30], i.e. conversion of forest land to agriculture occurs when the potential agricultural revenues from a piece of land exceed the costs of production and clearing the existing forest [31]. Several biophysical, socioeconomic and institutional factors may influence the costs or returns of converting forested land to agriculture. Forest clearing has been linked to several economic factors like expected prices and demands for commodities like palm oil [30], higher commodity prices and potential agricultural revenue [32]. Transportation costs are also expected to impact deforestation probabilities [30] and proximity to built infrastructure has been linked to greater deforestation [32], however, changes in transport costs have been shown to influence deforestation differently based on prior land use and extent of development [33]–they are typically represented using proxies like distances to roads, cities, markets, etc. Oil palm expansion, specifically, has been linked to elevation, slope, precipitation, soil type, distance to existing palm oil areas, as well as distance to palm oil extraction centers, roads, ports, population centers, and settlements [34–36]. Other accessibility and infrastructure measures such as distance to forest edges, communications cost, presence of protected areas and other land use designations or zoning criteria have also been shown to influence deforestation dynamics [29,30].

Most economic or infrastructure variables influencing expansion of oil palm plantations change with time as market prices, road networks, area under plantation, etc. change. Other variables like land use designations, protected areas, and other institutional factors also might potentially change, although, they are usually considered to be static due to their nature of being constant over long time periods. As the demand for commodities, land use dynamics, and availability of suitable land changes, it is likely that drivers of oil palm expansion will also change. With its long history of forest conversion and plantation cultivation, Peninsular Malaysia now has limited suitable land available for expanding oil palm plantations. Given the forest dynamics of Peninsular Malaysia are distinct from that in Malaysian Borneo, we assess the determinants of oil palm expansion specifically in Peninsular Malaysia and changes in these determinants over time.

Determinants and constraints are essentially two sides of the same coin and identifying determinants of land use change has been used to recognize and explore constraints to related land use changes [37]. Previous global studies have used suitability for oil palm to determine potential areas for oil palm expansion [38,18]. Malaysia has very high climate suitability for oil palm [39] and 88% of its land area is biophysically suitable for oil palm cultivation [40]. However, due to extensive land use change in the past, there is limited land available in Malaysia for future oil palm expansion [22]. With low land availability, other competing land uses, and various government designated land uses it is understandable that agro-environmental suitability alone cannot determine areas of oil palm expansion. Protected areas and other existing land uses have been considered to limit areas available for future expansion of oil palm [38,18]. High carbon stocks or areas of high conservation value have also been explored as possible constraints for future expansion when taking implementation of sustainability criteria into consideration [38]. Accounting for biophysical suitability, land already under use or protection and different sustainability criteria leaves very little of the biophysically suitable area available for expansion; only 17% of global palm oil suitable area would be available upon considering all the restricting criteria [38]. Sparing high carbon stock or high conservation value land from conversion is currently proposed as part of the sustainability commitment of the palm oil sector [38] and high conservation value areas are currently protected under the Roundtable on Sustainable Palm Oil (RSPO) regulations that apply to certified palm oil plantations producing 21% of the global palm oil [18]. However, they are either suggested sustainability criteria as a part of a proposed methodology [41] or criteria that aren’t applicable to majority of the forests and are not prohibitive and thus do not limit oil palm expansion by their very nature. Another constraint considered is the market accessibility of suitable areas and only 18% of the global suitable land is within 2 hours transportation to large cities [38]. Unlike high carbon or conservation value areas, infrastructure accessibility of a location on the other hand can be restrictive for future expansion as it might potentially influence the profits generated plantations in that location.

In order to better understand where future oil palm expansion can threaten forests and biodiversity habitat characterizing patterns of expansion and factors associated with expanding oil palm plantations is essential. An understanding of the determinants of recent plantation expansion and the environmental suitability of those areas is essential for better land use planning and policy making. The objectives of this study are to (a) identify primary determinants of forest conversion to oil palm plantations and their temporal shifts between 1988–2000 (pre-2000) and 2001–2012 (post-2000), (b) spatially characterize patterns of agro-environmental suitability and socio-economic variables including market and infrastructure accessibility for recent areas of oil palm expansion, and (c) characterize constraints on converting remaining forest to oil palm plantations.

## Materials and methods

### Study area

Our study focuses on Peninsular Malaysia. It is an equatorial region experiencing the southwest monsoon from May to September and the northeast monsoon from October to March. The average temperatures are between 25 and 35 °C and average annual precipitation ranges between 1500–5000 mm. It thus has ideal climatic conditions for growing oil palm, which requires annual precipitation between 1000–5000 mm/m^2^, average annual temperature between 18 and 38 °C, and four or fewer months with rainfall lower than 100 mm/m^2^ [38]. Forested areas of Malaysia have a high potential for agriculture and an estimated 146,000 square kilometers of Malaysia’s forests in 2001 were estimated to be suitable for oil palm cultivation [42].

**Fig 1:**
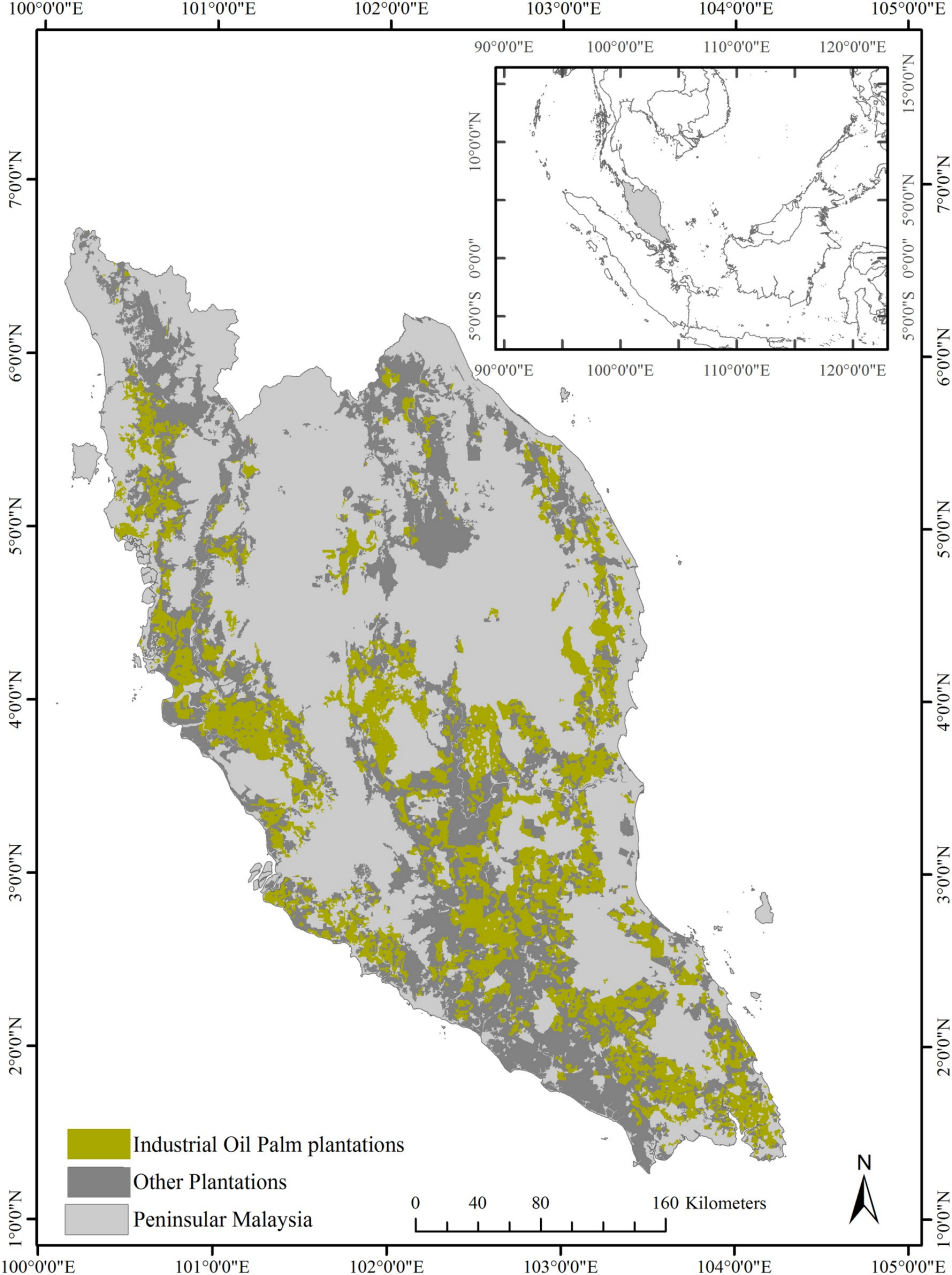
Peninsular Malaysia with industrial oil palm plantations and other plantations from World Resources Institute (WRI,) [44].

Peninsular Malaysia’s area under oil palm plantations increased from 1.7 Mha in 1990 to 2.7 Mha in 2010 [17]. Industrial plantations in peatlands continue to expand in the region with 276,000 ha of peatlands under industrial oil palm plantation in 2015 [43]. A recently generated plantation map [44] estimates Peninsular Malaysia had 5.5 Mha under plantations in 2014 and large industrial-sized plantations covered 2,631,070 ha with 89% (2,335,260 ha) under oil palm cultivation (Fig 1).

### Methodology

**Fig 2:**
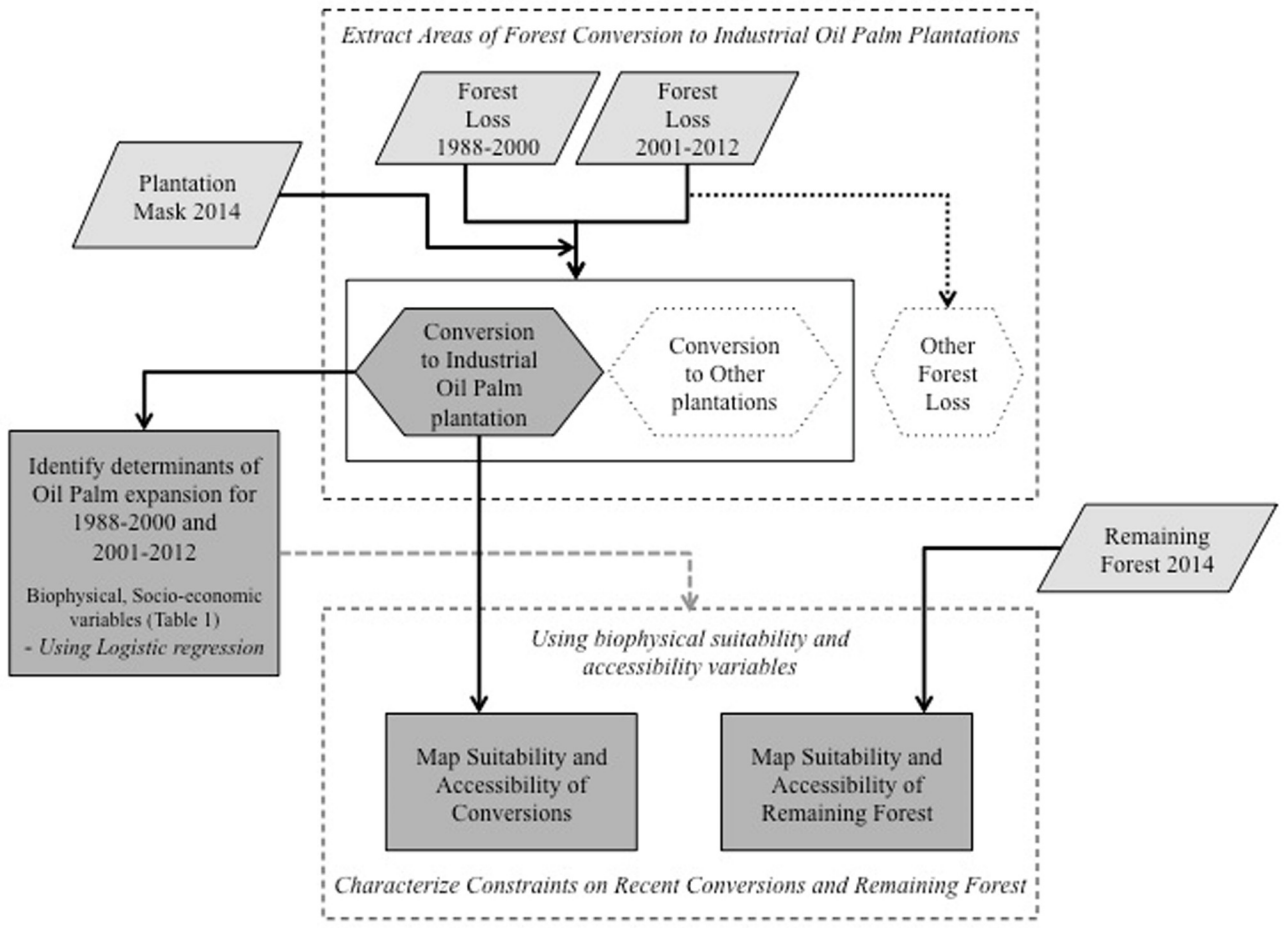
Overview of the methodology.

To achieve our primary objective of identifying the determinants of oil palm expansion, we built several statistical models to assess the combined impact of various potential drivers defined in the scientific literature within a spatially explicit modeling framework. To identify possible constraints on future conversion of forests we characterized agro-environmental suitability and accessibility of recently converted areas. The overall methodological flow is described in Fig 2. This approach was applied to two time periods–pre-2000 (1988–2000) and post-2000 (2001–2012) to compare the impact of various determinants and constraints over time.

#### Data and variables

We use the tree plantation dataset from WRI (available from www.globalforestwatch.com) [44] for the region that delineates four types of plantations in the year 2014; large industrial-sized plantations, small-sized mosaic plantations, medium-sized mosaic plantations and recently cleared or young plantations. The large plantations tend to be monocultures and the species information associated with them was used to identify large single-species plantations of oil palm. Mosaic plantations also have species information associated with them and are dominated by oil palm and rubber but these tend to be a mix of a variety of species. As mosaic plantations are a mix of several species interspersed with croplands and settlements these can pose difficulties to identifying determinants and constraints to expanding plantations of specific species and recently cleared / young plantations do not have species information associated with them. Hence, we focus only on industrial oil palm plantations.

Natural forest loss is defined as complete or nearly-complete tree cover loss within a 30 m Landsat pixel occurring within a natural forest mask of 1988 developed by Shevade et al. [10]. The natural forest mask obtained from [10] delineates mature naturally regenerated forests with a minimum area of 5 ha and was developed using a bagged decision tree model applied to multi-temporal metrics derived from a 1988 Landsat image mosaic to develop (see [10] for further details). Tree cover loss for 1988–2000 (pre-2000), obtained from Shevade et al. [10], was mapped in a one time-step (due to the limited availability of Landsat imagery before 2000) while 2001–2012 (post-2000) loss mapped annually was obtained from the Global Forest Change (GFC) product [7]. The tree cover losses for pre-2000 and post-2000 use similar methods providing a consistent forest loss dataset for the region. Using the natural forest mask for Peninsular Malaysia to identify loss within the existing natural forest overcomes the limitations of the GFC tree cover loss dataset, which does not distinguish between natural forests and plantations. Forest conversion is defined as all the natural forest loss (1988–2012) that is within plantation areas [44] and conversion to oil palm refers to forest loss converted to large industrial plantations of oil palm.

#### Factors influencing conversion to plantations

Several variables were evaluated as factors influencing deforestation and conversion to plantations. Variables chosen have been shown to influence deforestation, conversion to plantations or environmental suitability of oil palm and are listed in Table 1 below.

1. Agro-environmental factors: Environmental variables that determine suitability for oil palm or influence forest conversion to these plantations were used in the model. Topographic variables like elevation and slope not only determine agricultural suitability for oil palm [38] but also determine accessibility for deforestation and conversion [28]. Other environmental variables used in the model that determine suitability for oil palm include annual precipitation, and mean temperature [38]. Additionally, minimum precipitation (average yearly minimum precipitation derived from WorldClim [47]) was also used for the analysis.
2. Access to markets: Access to large cities is related to the accessibility of areas to markets and costs of transportation and has been used to model deforestation [30] or to assess the economic appeal of areas available for conversion [38]. Proximity to urban areas has been also associated with higher deforestation [32]. Given palm oil is traded internationally, distance to major ports is expected to influence forest clearing [30] and has been shown to negatively influence oil palm expansion in Indonesia [36]. We used the following major Federal ports—Johor Port Pasir Gudang, Kemaman Port, Kuantan Port, Penang Port, Port Klang, and Port Tanjung Pelepas and calculated cost distance to ports (using roads obtained from: http://sedac.ciesin.columbia.edu/data/set/groads-global-roads-open-access-v1 to calculate distance to roads) for our analysis [45].
3. Access to infrastructure: a) Distance to previously established oil palm plantations: Previously established plantations have been shown to heavily influence newer areas of conversion to plantations [29,35]. Old deforestation / plantations and areas around them will have established infrastructure like cleared areas, access roads, and settlements for workers, making it easier to develop newer plantations adjacent to those lands and reducing costs of conversion. “Old” plantations are assumed to be plantations existing before the study period in question. We used the WRI plantation mask for industrial oil palm plantations and removed all plantation areas converted from forests between 2000 and 2012 to get plantations existing before year 2000 and removed all plantation areas converted from forests between 1988 and 2012 to obtain plantations existing before 1988. As detailed trajectories of land use change are not available for the region, we assumed that all areas deforested prior to 1988 and that are currently under oil palm cultivation, were converted to oil palm plantations immediately following clearing. Euclidian distances to these “old” plantations–plantations that were already established by the time period under consideration—are then calculated. b) Distance to palm oil-processing facilities: Access to palm oil-processing mills has been shown to explain presence of oil palm plantations [34]. Palm oil mills within Peninsular Malaysia obtained from WRI. c) Distance to settlements: Proximity to settlements has been shown to be preferred for expansion of oil palm plantations [35]. Settlements within the region were obtained from Socio Economic Data Acquisition Center (SEDAC) (Available from http://sedac.ciesin.columbia.edu/data/set/grump-v1-settlement-points).
4. Socioeconomic Factors: Population has been linked with greater deforestation in several studies [32].

**Table 1:** Explanatory variables.

	Variable	Source	
	Total Precipitation (mm)	WorldClim (30 arc seconds) http://worldclim.org/version2 [47]	
Climatic variables	Minimum Precipitation (mm)		
	Mean Temperature (Celsius)		
Topographic variables	Elevation (m)	NASA Shuttle Radar Topography Mission (SRTM) (90m)	
	Slope (degrees)		
Accessibility variables *	Distance to Old Plantations (km)	WRI [44] www.globalforestwatch.com	Calculated Euclidean distance to source
	Distance to Palm Oil Mills (km)		
	Distance to Settlements (km)	SEDAC http://sedac.ciesin.columbia.edu/	
	Distance to Major ports (km)	Google Earth	
	Accessibility to large cities (population >50,000) (travel time in min)	Joint Research Centre of the European Commission http://forobs.jrc.ec.europa.eu/products/gam/download.php (30 arc seconds) [48]	
Socioeconomic	Population (density per km^2^) in 2000 / 2010	SEDAC http://sedac.ciesin.columbia.edu/ (1km) [46]	

* Models used logs of all the accessibility variables.

### Identifying determinants of oil palm expansion

To identify determinants of oil palm expansion, we modeled forest conversion to oil palm plantations for pre-2000 and post-2000 separately. We used pre-2000 and post-2000 forest conversion to oil palm plantations separately to generate our presence (forest conversion to oil palm) and absence (persistent forest) samples for forest conversion for each of the models using an equalized stratified random sample of a total 500 points. We used logistic regression models as these are well-suited for binary dependent variables and have been used successfully for predicting land cover change [28, 32, 36, 49]. We related the presence or absence of conversion to oil palm (binary dependent variable) with the explanatory variables (Table 1) extracted at the sample points, using logistic regressions for the two study periods separately. To avoid the effect of multi-collinearity, we selected independent variables after testing for pairwise correlations and selected only one variable from a pair when the Pearson’s correlation coefficient was greater than |0.7| for any pair of variables. As mean temperature and elevation were highly correlated (Pearson’s correlation coefficient -0.97) we decided to retain only elevation for the logistic regression.

Logistic regression analysis (using the GLM package in R) was followed by hierarchical partitioning (using the hier.part package in R) to assess the contribution of individual variables to the model by calculating the percentage of total variance explained by the independent variables [50]. We classified the pre-2000 model predicted probabilities into two classes of ‘predicted oil palm’ and ‘predicted forest’ using a probability threshold of 0.9. We used this map of predicted oil palm / forest to assess model accuracy using forest loss converted to oil palm plantations between 2001 and 2012.

### Characterizing patterns of recent oil palm expansion

Agro-environmental suitability for a crop is considered an important factor for its cultivation in any particular area. We used biophysical suitability classification for oil palm production by Pirker et al [38] (Available for download from: http://www.iiasa.ac.at/web/home/about/news/160722-Palm_Oil.html) to determine the biophysical suitability of the pre-existing plantations (pre-1988) and recently converted (1988–2012) oil palm plantations within the region. We also used the suitability classes to determine the biophysical suitability of recently converted oil palm plantations.

We used accessibility to large cities, accessibility to palm oil processing, and accessibility to existing oil palm plantations (per study period) to determine market and infrastructure accessibility for all pre-existing plantations (pre-1988) and recently converted (1988–2012) oil palm plantation areas. Accessibility of recent conversions to large cities in travel time [48] was used as a proxy to determine their accessibility to markets and transportation costs. Lower travel time represented greater accessibility to markets. We classified accessibility to cities into 6 categories; < 1hr, 1–3 hrs, 3–6 hrs, 6–12 hrs, 12–24 hrs, and >24 hrs. Accessibility to palm oil mills for processing is an important factor influencing the establishment of oil palm plantations and distance to palm oil mills was used as a proxy for accessibility. Distance to palm oil mills was classified into 5 categories; < 5km, 5–10 km, 10–20 km, 20-40km and > 40km. Distance to previously existing plantations was used to determine accessibility to old plantations. Accessibility to old plantations was classified into 5 categories; < 1 km, 1–2 km, 2–4 km, 4–8 km, > 8 km.

To understand the characteristics of areas with low agro-environmental suitability for oil palm that might lend to establishment of oil palm plantations, we determined the market and infrastructure accessibility of all areas of forest loss (pre-2000 and post-2000) converted to oil palm plantations. We used the marginal suitability and moderate suitability for oil palm from Pirker et al [38] to define low suitability for oil palm.

### Characterizing constraints on future conversion of forests to oil palm plantations

We characterized agro-environmental suitability and accessibility constraints to future forest conversion to oil palm plantations. The remaining natural forest in 2014 for Peninsular Malaysia was determined using the natural forest mask for circa 1988 and subtracting all the areas of mapped forest loss between 1988 and 2000 (from [10]) and 2000–2014 (from [7] updated for year 2014). To map the constraints onto the natural forest (2014) we used the classification for suitability as described by Pirker et al [38] and classifications for accessibility as described in the previous section. Additionally, restrictions on conversions due to the protection status, land ownership, presence of peat soils, etc. will also impact where future conversions take place and have been considered as constraints in other studies [18, 38]. We considered remaining forest within protected areas and the presence of peat soils as possible constraints. We used the following protected areas for our assessment, Taman Negara National Park (434,300 ha), which is the largest and oldest National Park in the region, Endau Rompin National Park, Royal Belum State Park, and Krau Wildlife Sanctuary [51].

## Results

### Determinants of plantation expansion

Our logistic regression models for forest conversion to industrial oil palm plantations identify a combination of environmental variables and socio-economic variables as significant (Table 2). Distance to old oil palm plantations is an extremely significant variable (p < 0.001) having a negative effect on forest conversions to oil palm plantations during both time periods (pre-2000 and post-2000) and could be considered the most important determinant of forest conversion to oil palm plantations.

**Table 2:** Logistic regression results for pre-2000 and post-2000 models.

	Pre-2000 model			Post-2000 model		
	Estimate	Std. Error	Pr(>|z|)	Estimate	Std. Error	Pr(>|z|)
**(Intercept)**	-7.785	5.254	0.138	1.497	5.977	0.802
**Elevation**	-0.007	0.006	0.289	-0.003	0.007	0.600
**Slope**	-0.233	0.095	0.014*	-0.049	0.099	0.619
**Total Precipitation**	-0.002	0.001	0.180	0.003	0.002	0.083.
**Minimum Precipitation**	0.080	0.026	0.002**	-0.077	0.029	0.009**
**Population**	-0.009	0.005	0.096 .	-0.003	0.004	0.422
**Accessibility to Large Cities**	0.901	1.082	0.405	0.557	0.931	0.550
**Distance to Major Ports**	0.139	0.403	0.730	0.474	0.304	0.119
**Distance to Palm Oil Mills**	-0.117	0.534	0.827	0.355	0.857	0.679
**Distance to old Oil Palm Plantations**	-3.000	0.463	0.000***	-3.665	0.744	0.000***
**Distance to Settlements**	-0.317	0.877	0.718	-2.682	1.283	0.037*

Notes: Significance levels of raw coefficients are shown as: 0 ‘***’ 0.001 ‘**’ 0.01 ‘*’ 0.05 ‘.’ 0.1

**Fig 3:**
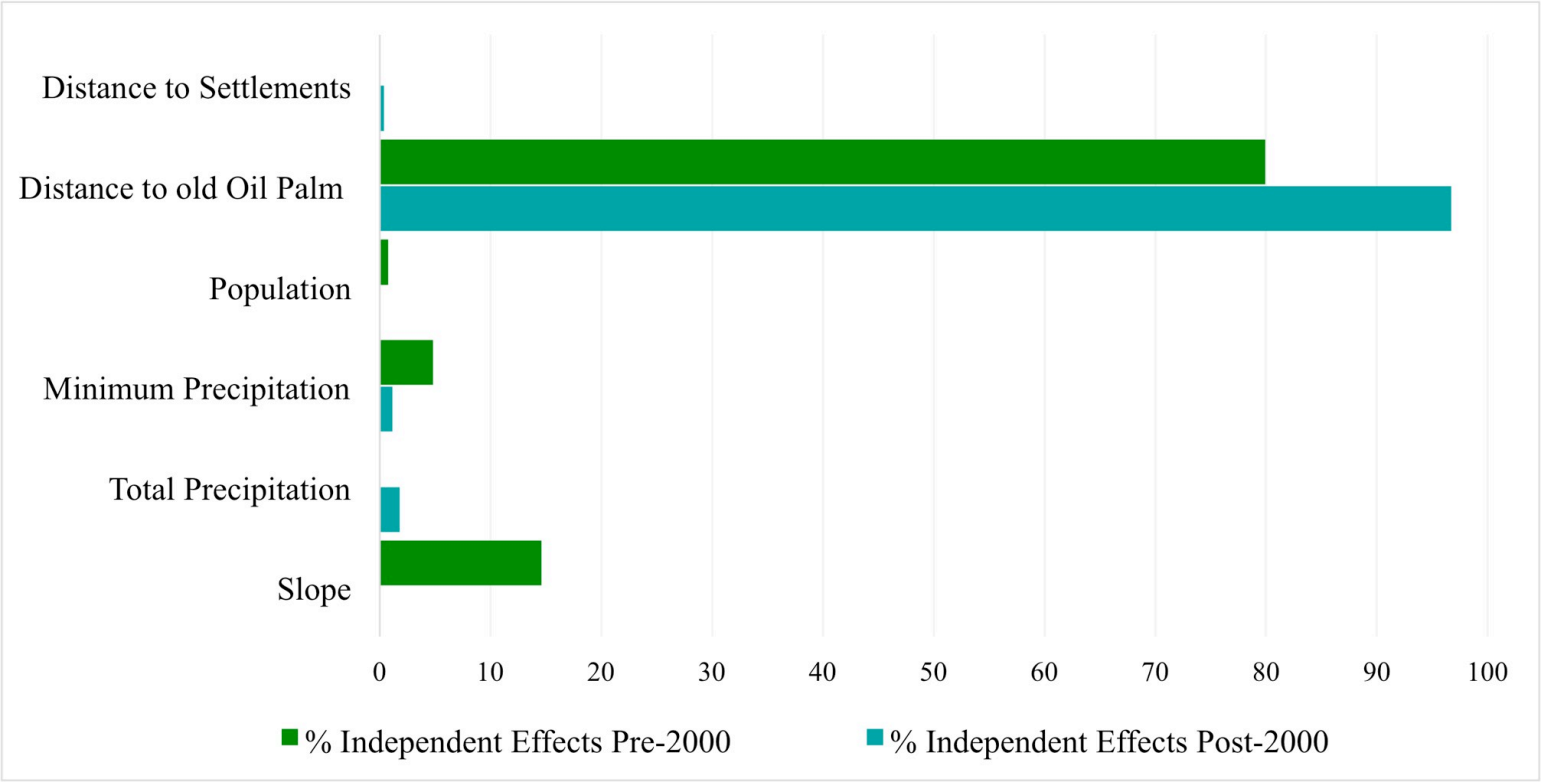
Independent contributions of significant variables for pre-2000 and post-2000 using hierarchical partitioning.

For the pre-2000 model, slope and population density have a significant negative effect while minimum precipitation has a significant positive effect on forest conversions to oil palm. For the post-2000 model, minimum precipitation and distance to settlements have significant negative effects on forest conversions to oil palm. Minimum precipitation changes from being positively significant for pre-2000 conversions to being negatively significant for post-2000 conversions. Additionally, population has a small significant (p < 0.1) negative effect for pre-2000 conversions and total precipitation has a small significant (p < 0.1) positive effect for post-2000 conversions. The independent contributions of the variables are shown in Fig 3., Distance to previous plantations had the greatest contribution for both pre-2000 (79.9%) and post-2000 (96.6%) models.

The overall accuracy of the pre-2000 model is 98.25%. The producer’s accuracy for ‘predicted oil palm’ class is 77.22% while the user’s accuracy is 59.17%. The lower user’s accuracy corresponds to a higher commission error, which is expected when predicting forest conversions to plantations without a suggested timeline for the predicted changes [28]. In addition, the lack of information on land tenure and the possibility or legality of forest conversions in the model also affect predictions of where forest conversion to oil palm will occur in the future.

### Suitability and accessibility characterization of recent conversions

Pirker et al [38] define 5 classes of biophysical suitability for oil palm cultivation primarily determined by low slopes and elevations and moderate to high rainfall; marginal, moderate, suitable, high and perfect. We used their biophysical suitability classification to assess the suitability of forest conversions to oil palm since 1988. Majority (76%) of Peninsular Malaysia is within high suitability classes (suitable, high, perfect) for oil palm. A large area of Peninsular Malaysia was already under plantations by 1988 and almost all (93%) of the pre-1988 plantations that were under oil palm (in 2014) have predominantly been established in areas within high biophysical suitability for the species. Thus, by 1988 only 56% of the natural forests were within high biophysical suitability for oil palm cultivation.

**Fig 4:**
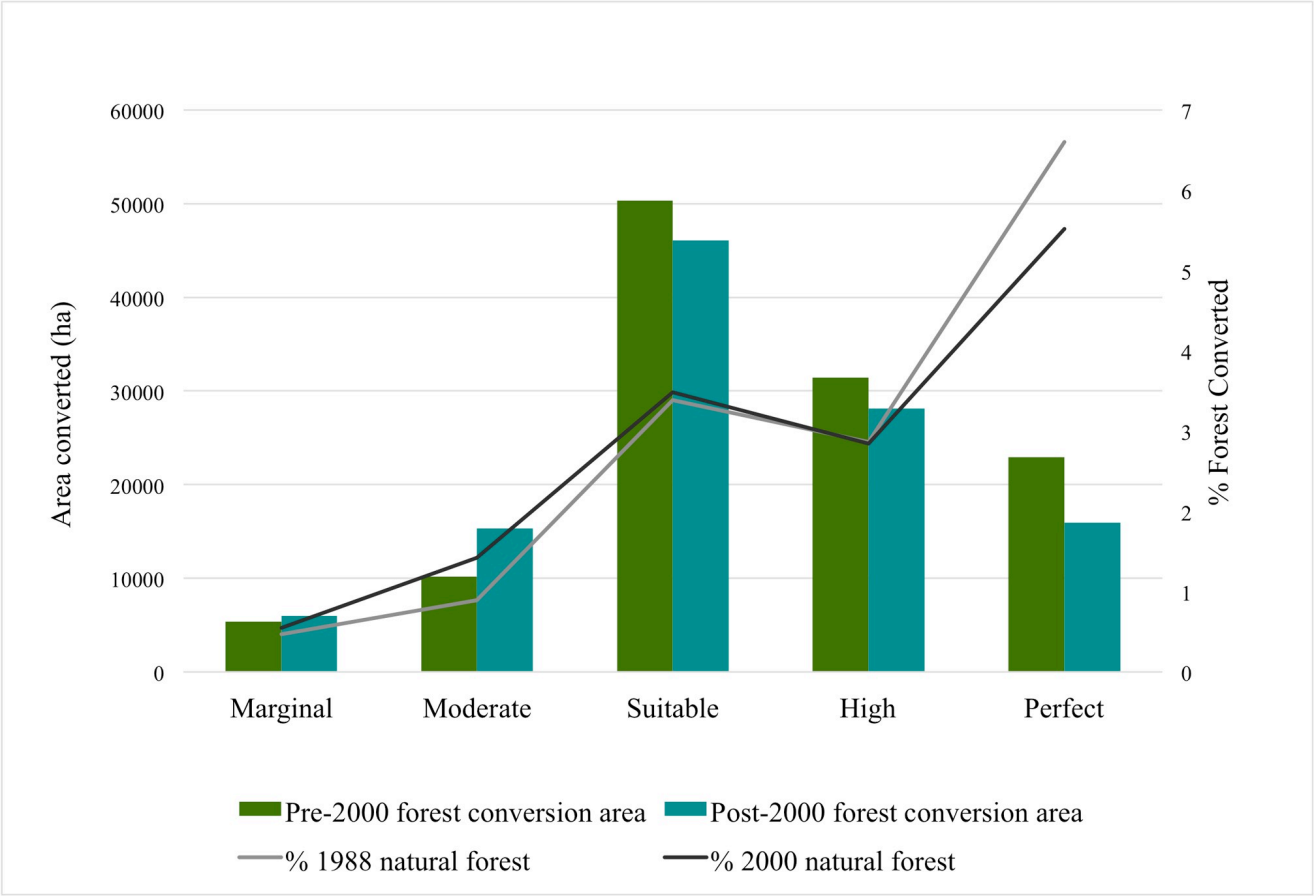
Pre-2000 and post-2000 area and percent area of natural forest converted to oil palm plantations by suitability class.

**Fig 5:**
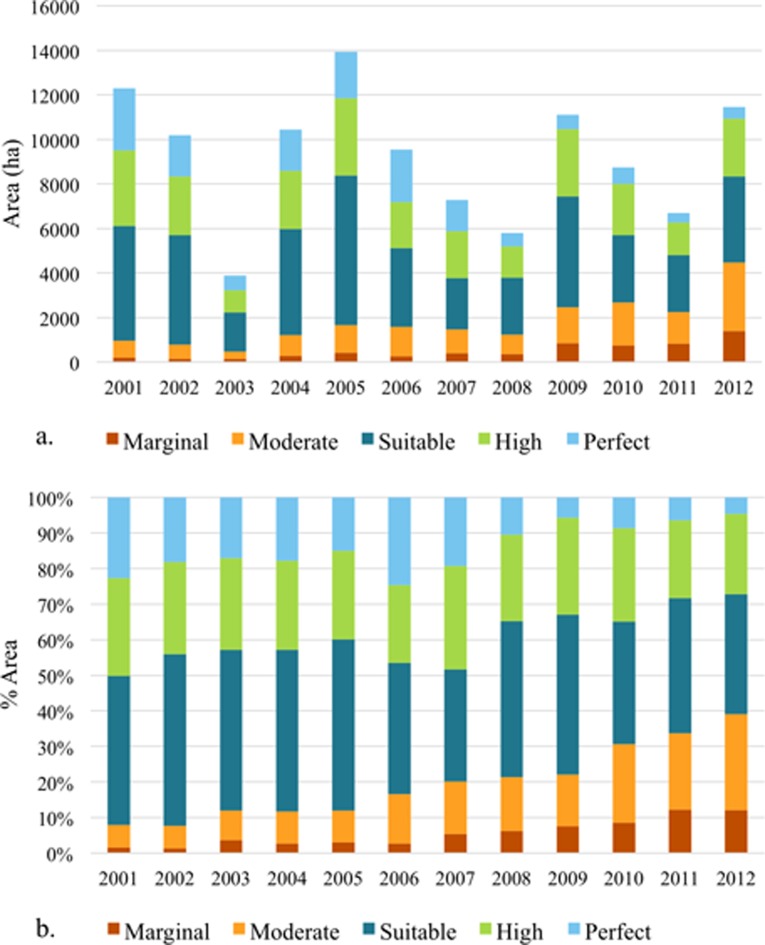
a. Yearly (2001–2012) area of forest conversion to oil palm by suitability class and b. Yearly proportional forest conversion by oil palm suitability class.

Of all the forest area converted to oil plantations between 1988 and 2014, most (> 80%) conversions have taken place within areas with high suitability for oil palm cultivation (Fig 4). There has been an increase in the proportional forest conversions to oil palm plantations in the low suitability classes (marginal and moderate). There is also a small increase in the absolute area and percent area of available forest conversions to oil palm within low suitability classes from pre– 2000 to post– 2000. This is evident when considering the yearly (between 2001 and 2012) forest conversions to oil palm plantations (Fig 5). Although the total area of conversions has fluctuated throughout the period, we find that both the absolute and proportional area of forest converted within marginal and moderate suitability classes has been increasing throughout the decade (especially since 2006, Fig 5a and 5b) while that in the higher suitability classes has been reducing.

**Fig 6:**
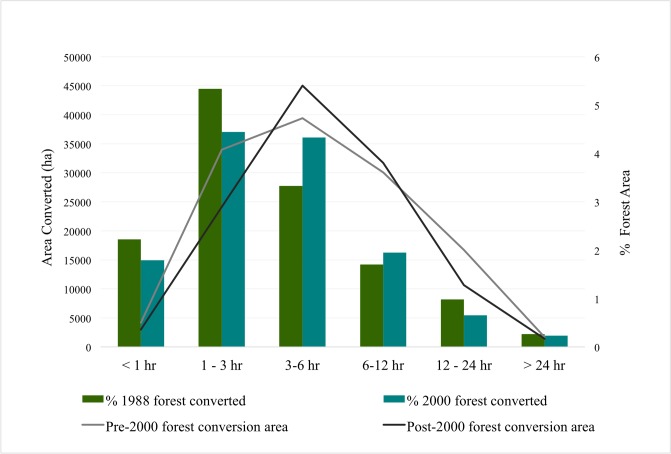
Pre-2000 and post-2000 forest area and percent forest converted to oil palm plantations by accessibility to large cities (travel time in hours).

Forest conversions to oil palm have been in regions with very high accessibility to infrastructure. All pre-2000 and post-2000 forest loss that was converted to oil palm plantations was in regions with very high accessibility to old oil palm plantations. Almost all (99.85% of pre-2000 forest loss and all of the post-2000 forest loss) that is now under oil palm cultivation was within 1km from pre-existing oil palm. Pre-2000 and post-2000 absolute and percent area of forest conversion to oil palm was greatest in regions with moderate accessibility to cities and reducing with decreasing accessibility to cities (Fig 6). Areas within 1 hour of cities also had low forest conversion to oil palm. Percent forest area converted is greatest at moderate travel times to large cities and reduces beyond that where forest converted is reduced drastically at travel times greater than 12 hours and becomes negligible at travel times greater than a day.

**Fig 7:**
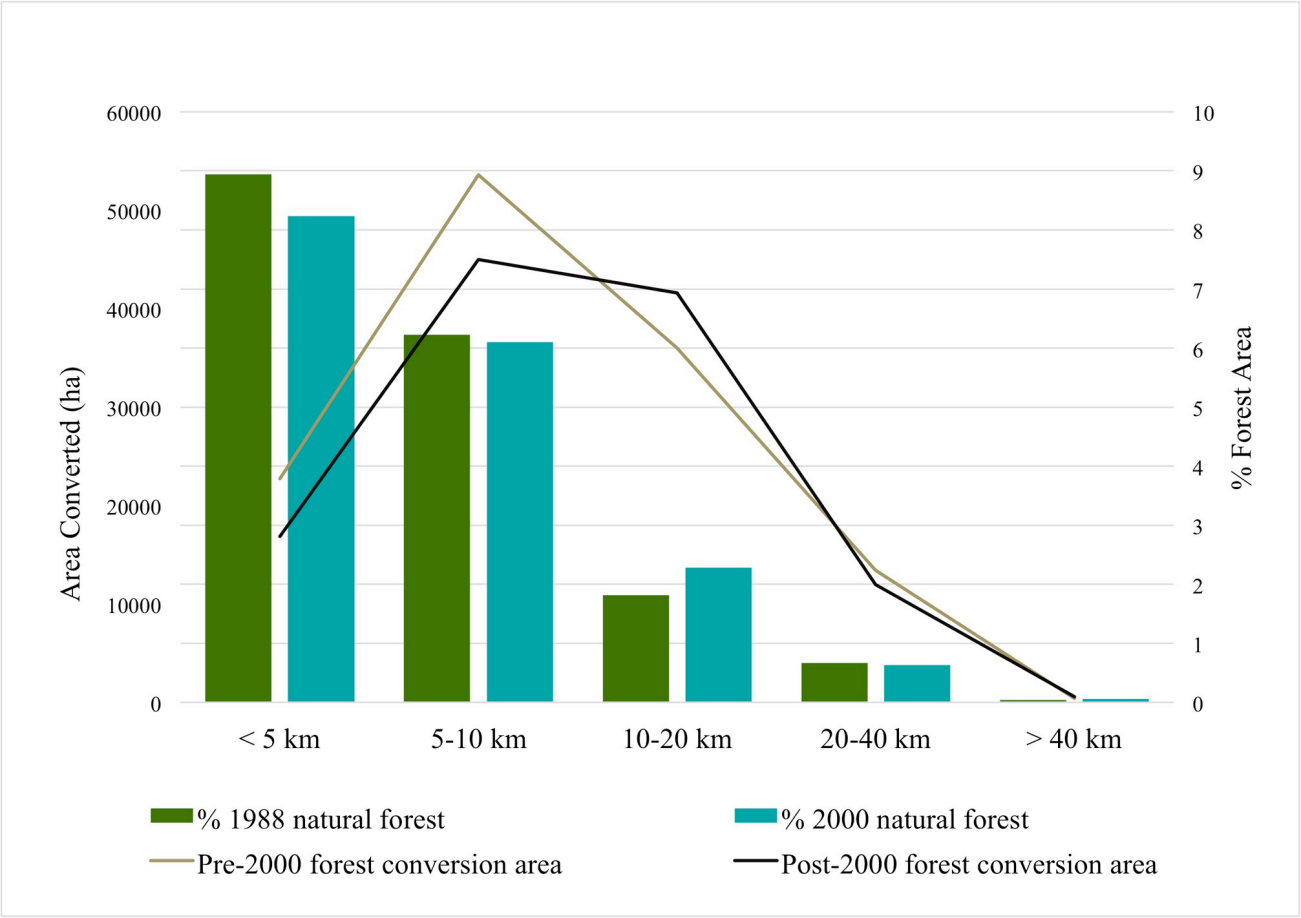
Pre-2000 and post-2000 forest area and percent forest area converted to oil palm plantations by proximity to palm oil processing centers.

Area of forest converted to oil palm is lowest in areas closest (< 5km) to palm oil processing mills, peaks at moderate distances between 5km and 10km of the mills before reducing with greater distances (Fig 7). The percent forest area converted to oil palm reduces with decreasing accessibility to oil palm processing facilities. These patterns are similar for both pre-2000 and post-2000 forest conversions.

Furthermore, 50% of the increase in conversions within low suitability classes between pre-2000 and post-2000 is a result of increased conversion in areas with high accessibility to palm oil mills. Conversion within areas of low suitability was predominantly in areas with high infrastructure accessibility. About 74% of pre-2000 forest conversions and at least 78% of post-2000 conversions were within areas moderate to very high accessibility to cities, had moderate to high proximity to palm oil processing mills and were all also within 2 km from previously existing oil palm plantations.

### Constraints on remaining natural forest areas (2014) for oil palm expansion

**Fig 8:**
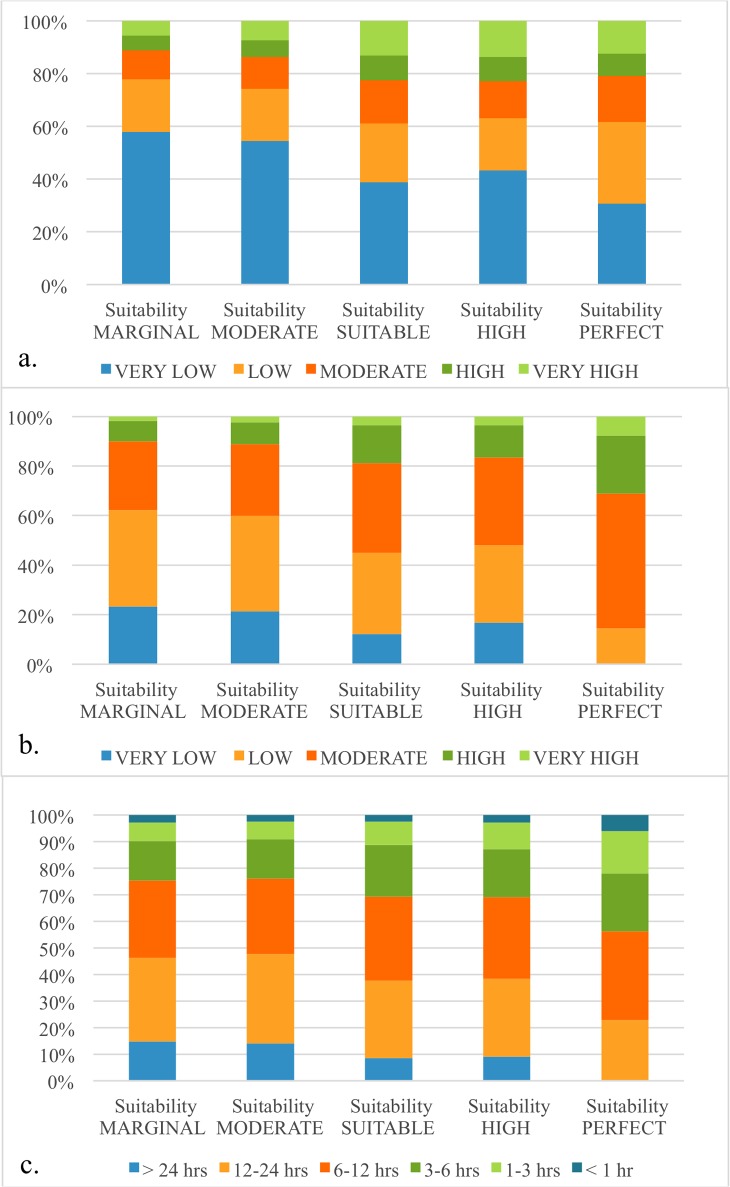
Remaining natural forest (2014) in biophysical suitability classes for oil palm separated by a) proportional accessibility to old oil palm plantations, b) proportional accessibility to palm oil mills and, c) proportional accessibility to large cities.

**Fig 9:**
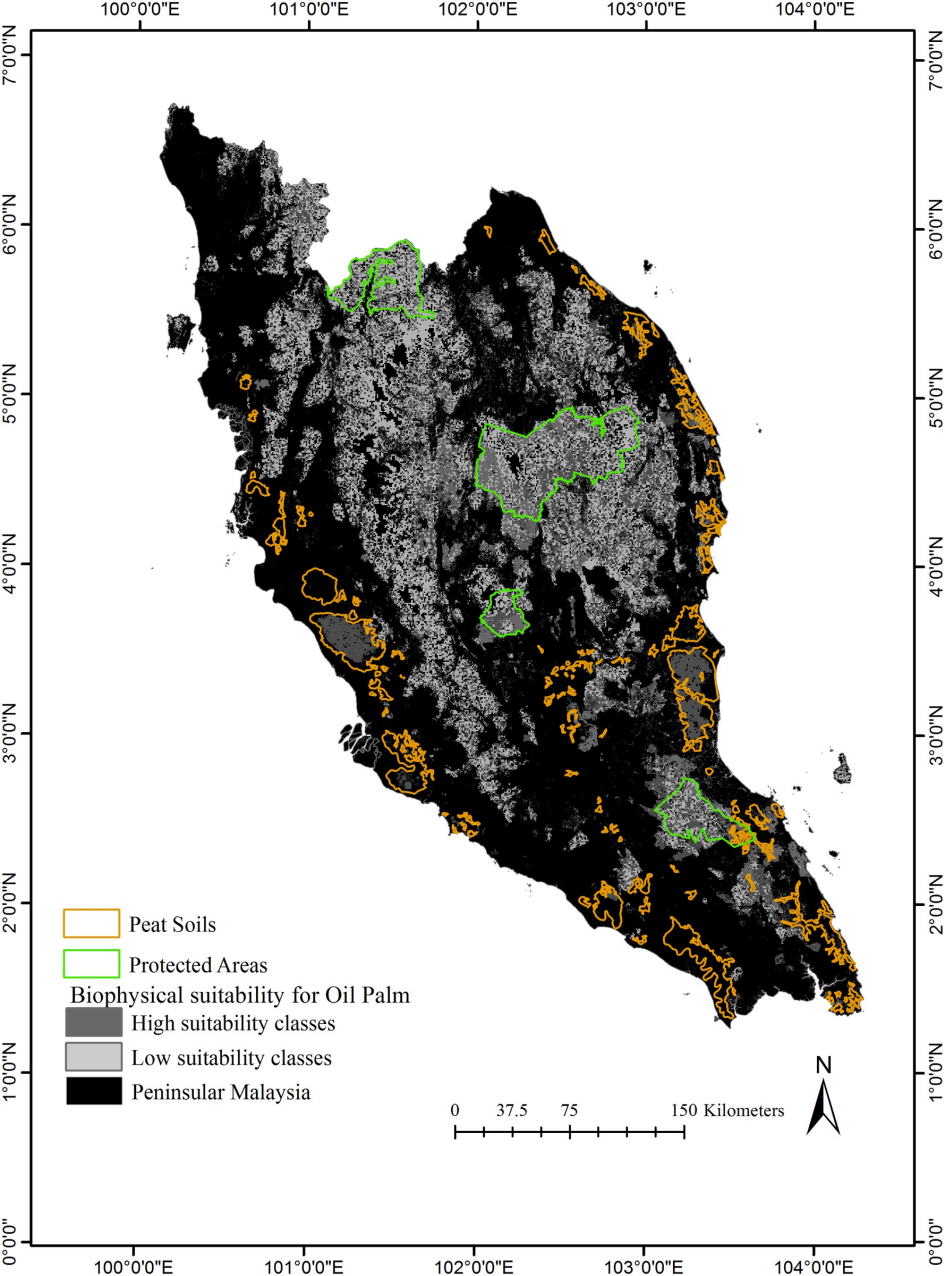
Remaining natural forests (2014) within high and low suitability classes for oil palm, protected areas from the World Database on Protected Areas (WDPA) [52] and on peat soils [53].

Approximately half (~52%) of the remaining natural forest in 2014 is well suited (suitability class–suitable, high or perfect) for oil palm cultivation and about 57% has moderate to high market accessibility. Of the remaining forest only 31% has moderate to very high accessibility to old oil palm plantations and only 22.5% is within 5 hours travel time of large cities (Fig 8a and 8c) while about 48% of the remaining forests have moderate to very high accessibility to palm oil processing (Fig 8b). About 31% of remaining natural forest with low suitability and 40% of the forest with high suitability for oil palm cultivation is within the four protected areas and 7% of the total remaining natural forest under high / perfect suitability is in peat swamps (Fig 9).

## Discussion

Peninsular Malaysia’s oil palm plantations have been primarily established in coastal and low-lying areas, which are biophysically highly suitable for oil palm production. As a result, most high suitability areas within the region have already been converted. However, conversions within marginal and moderate oil palm suitability classes have been increasing and about 30% of all conversions in 2012 were in areas with low oil palm suitability. The increased conversions in low suitability areas might be driven by the limited land availability in areas with high suitability for oil palm and have been observed in Sumatra [54]. With low land availability, competing land uses and continued increase in the global palm oil demand, this recent expansion of oil palm plantations in low suitability areas might indicate economic and infrastructure factors are driving the establishment of oil palm rather than strictly biophysical factors as has been discussed by Sayer et al [55].

Indeed, our regression analysis and characterization of forest conversions from 1988–2012 also indicate that accessibility of a location to existing infrastructure, especially pre-existing plantations is more important than its biophysical characteristics for oil palm cultivation and that lack of accessibility has constrained forest conversions to industrial oil palm plantations. Slope and minimum precipitation are the biophysical factors that are associated with oil palm establishment. Slope, however, does not have a significant effect on post-2000 conversions. Minimum precipitation changes from having a significant positive effect on conversions before 2000 to having a negative effect on conversions after 2000. This could indicate that older conversions were in areas with more rainfall while more recent conversions have been in areas with lower rainfall. As the best available land had already been converted, this suggests expansion is moving into more marginal lands as has been observed since 2006.

The importance of connectivity and infrastructure with respect to expansion of oil palm plantations has been observed previously in Indonesia [20, 36], where oil palm plantations are located in areas with easy access and lack of proximity to existing infrastructure like roads, concessions, plantations, and ports reduces the likelihood of oil palm expansion [36]. In Colombia and Latin America too, oil palm plantation expansion is often clustered and is most likely in areas closest to existing plantations [34, 56]. The importance of proximity to old plantations for conversions might indicate that economic incentives play a greater role than and at times override the influence of agro-environmental suitability of a region for oil palm establishment such that profits generated despite low suitability are greater than costs. Farmer perception surveys in Indonesia have informed that planting of oil palm is constrained by lack of accessibility of villages, which is eventually overcome with road development and changes in the landscape [57] rather than rejection of the crop. The relatively lower importance of biophysical characteristics for oil palm establishment could also be a result of highly intensive plantation management, use of irrigation, or use of improved cultivars that could overcome poor biophysical suitability for oil palm crops. Additionally, the generalized nature of the input datasets used for generating the biophysical suitability map, like the climatic information, which has a lower quality in tropical areas and the soil data, which has a lower reliability in South Asia, limit the reliability of the biophysical suitability map [38] and might result in misclassification of some areas.

Although oil palm has been planted successfully in regions with elevation of up to 1500m and slopes up to 16 degrees [38], Harris et al [58] have observed that 96% of oil palm existing in parts of southeast Asia by 2010 were established at elevations below 200m and that 65% of these plantations were on slopes below 3% rise. The suitability classification [38] uses very broad topographic criteria allowing elevations up to 1500m and slopes up to 25 degrees in suitability classes, which might not be very realistic for the region. It is important to understand this limitation of the analysis. Most of the remaining forest area within marginal or moderate suitability classes (~77%) either is at higher elevations (>300m) or has steeper slopes (>16 degrees) making it less accessible and less likely to be converted. Changes to the biophysical characteristics like the loss of soil fertility can also influence future conversions. Soils in oil palm plantations in Sumatra have been extensively degraded as a result of management, erosion, compaction over time and lack of carbon inputs [54]. This was observed especially in older plantations, which have been typically planted on land previously used for plantations like rubber or in marginal areas due to land scarcity [54]. The long history of plantation cultivation in Peninsular Malaysia and the more recent expansions into marginal lands can also result in soil degradation in the region.

Although the presence of peat has been considered a constraint, it is not necessarily a deterrent to forest conversion. Peatlands have been drained and converted in the past and an estimated 27% of peatland forests in Peninsular Malaysia had been converted to large oil palm monocultures by the early 2000s [13]. By 2015, about 31% of peatland area in Peninsular Malaysia was converted to industrial plantations with a majority of these under oil palm plantations (98.7%, 275,680 ha) [43]. Similarly, all protected areas are also not necessarily effective in preventing forest loss and conversions and protected area effectiveness does vary with the protection status [59]. In the four protected areas considered, we observed forest loss of 12258 ha from 2001–2016, mostly concentrated in the southern part of Endau Rompin. This suggests that presence of protected areas as a constraint for conversion might depend on its protection status. Additional constraints on conversion to oil palm plantations could include local land tenure and ownership of land, state and federal policies for development, market demand and commodity prices, and other competing land uses, for example about 80% of Peninsular Malaysia’s forests under Permanent Reserved Forests (PRFs) are designated for logging [60].

It is important to note that not all factors associated with oil palm cultivation are static in nature. Proximity to palm oil processing, existing plantation infrastructure, and markets are all potentially highly dynamic variables. The accessibility of a location to infrastructure will change with new roads, processing mills or new plantations or as new trade centers emerge. Thus, any of these dynamic variables constraining oil palm cultivation at a location in the present might not continue to be constraints in the future or there could be new constraints in the future, for example suitability for oil palm in Malaysia is expected to change drastically with the climate; studies project large reductions in areas with highly suitable climate but increases in suitable and marginal climate area by 2100 [39, 61]. Finally, the assessment of determinants and constraints is limited by the quality of the datasets used for this analysis. Errors in the forest / forest loss datasets, plantation boundaries or missed plantation areas will impact the estimates of conversion and possibly the estimation of determinants and constraints.

As has been suggested previously [18, 36, 38], forest conservation incentives should be designed to take into consideration the areas of potential oil palm expansion. Protected areas tend to be in areas with lower pressures and are less likely than average to be deforested [62]. Our results show that biophysical suitability alone is not sufficient to determine where future expansion can take place and accessibility to infrastructure should be considered. Accessibility to old oil palm plantations is strongly associated with conversion, ~8% of the remaining natural forest in 2014 within all suitability classes that is within 1 km of existing oil palm plantations could be vulnerable to conversion in the near future. Areas of high suitability and high accessibility are of greatest concern with regards to potential conversion to industrial oil palm. Areas of low suitability but high accessibility will have the next greatest vulnerability to conversion. Such areas with high biodiversity or conservation value or aboveground carbon / peat content should be prioritized for protection over other high conservation value areas with low accessibility. As previously shown, protected areas have a greater impact when they are established in areas closer to roads, cities and on lower slopes [62].

## Conclusion

Our study identifies proximity of previously existing oil palm plantations has been the strongest determinant of forest conversion from 1988 to 2012. Expansion of industrial oil palm plantations replacing forests between 1988 and 2012 has predominantly (> 99%) been within 1 km from oil palm plantations that have been established earlier. Given the perfect environmental conditions for growing oil palm in Malaysia and a large proportion of its land area having high biophysical suitability for the crop, most previously established plantations have been in regions of high biophysical suitability. With extensive land already under plantation cultivation and limited land availability, only 5% of forest “perfectly suitable” for oil palm remains. However, greater conversions in regions with low oil palm suitability since 2006 suggests that low suitability is not necessarily a limiting factor for recent forest conversions to industrial oil palm in the region. As most of the expansion of plantations has been in areas adjoining existing oil palm plantations, we determine that poor accessibility to infrastructure is the most important constraint to future forest conversions to oil palm plantations in Peninsular Malaysia.
